# Modulation of Macrophage Functional Polarity towards Anti-Inflammatory Phenotype with Plasmid DNA Delivery in CD44 Targeting Hyaluronic Acid Nanoparticles

**DOI:** 10.1038/srep16632

**Published:** 2015-11-18

**Authors:** Thanh-Huyen Tran, Ruchir Rastogi, Juili Shelke, Mansoor M. Amiji

**Affiliations:** 1 Department of Pharmaceutical Sciences, School of Pharmacy, Bouve College of Health Sciences, Northeastern University, Boston, MA 02115.

## Abstract

The purpose of this study was to modulate macrophage polarity from the pro-inflammatory M1 to anti-inflammatory M2 phenotype using plasmid DNA (pDNA) expressing interleukin-4 (IL4) or interleukin-10 (IL10)-encapsulated in hyaluronic acid-poly(ethyleneimine) (HA-PEI) nanoparticles (NPs). The HA-PEI/pDNA NPs with spherical shape, average size of 186 nm were efficiently internalized by J774A.1 macrophages. Transfection of HA-PEI/pDNA-IL4 and HA-PEI/pDNA-IL10 NPs increased IL4 and IL10 gene expression in J774 macrophages which could re-program the macrophages from M1 to M2 phenotype as evidenced by a significant increase in the Arg/iNOS level, and upregulation of CD206 and CD163 compared to untreated macrophages. Following intraperitoneal (IP) injection to C57BL/6 mice, HA-PEI NPs effectively targeted peritoneal macrophages over-expressing CD44 receptor. In an *in vivo* model of stimulated peritoneal macrophages, IP administration of HA-PEI/pDNA-IL4 and HA-PEI/pDNA-IL10 to C57BL/6 mice significantly increased the Arg/iNOS ratio and CD163 expression in the cells. Furthermore, HA-PEI/pDNA-IL10 NPs significantly increased peritoneal and serum IL10 levels which effectively suppressed LPS-induced inflammation by reducing level of TNF-α and IL-1β in peritoneal macrophages and in the peritoneal fluid. The results demonstrated that pDNA-IL10-encapsulate HA-PEI NPs skewed macrophage functional polarity from M1 toward an anti-inflammatory M2 phenotype which may be a promising platform for the treatment of inflammatory diseases.

Macrophages are key players of the immune system involving in phagocytosis, antigen presentation, and secretion of various cytokines, chemokines, and growth factors that protect the body from inflammation or infection^1^,^2^. They are highly plastic cells that can rapidly undergo morphological, functional, and biochemical changes in response to local environmental stimuli. In the presence of inflammatory stimuli, macrophages polarize toward pro-inflammatory M1 phenotype that produce high level of inflammatory cytokines and chemokines to eliminate pathogens^3^. In contrast, would healing environment promotes macrophage polarization to anti-inflammatory M2 state with increased production of anti-inflammatory cytokines, leading to alleviating inflammation, tissue repair and remodeling^4^,^5^. Emerging evidence has shown that recruitment of a large number of M1 inflammatory macrophages to inflamed tissues with high production of pro-inflammatory cytokines which contribute to inflammatory responses and tissue damage in inflammatory diseases^6^. Therefore, modulation of macrophage phenotype from an M1 to M2 state may be a promising approach for the treatment of inflammatory diseases.

Peritoneal macrophages are the most abundant cells among variety of immune cell populations reside in the mouse peritoneal cavity^7^, which play important roles in the defense against infection in the peritoneal cavity such as acute pancreatitis by releasing inflammatory mediators^8^,^9^. Recent studies have suggested that peritoneal macrophages are able to undergo systemic migration and recruitment to inflammatory sites^10^,^11^. Howard *et al*. demonstrated that local knockdown of TNF-α in peritoneal macrophages by intraperitoneal (IP) administration of chitosan/small interfering RNA (siRNA) nanoparticles inhibited inflammation and joint destruction in arthritic mice^11^. Likewise, Fellowes *et al*. reported that peritoneal macrophages were transfected by IP injection of human IL-10 expressing plasmid DNA-encapsulated cationic liposomes, subsequently the plasmid DNA was detected in the inflamed paws of collagen-induced arthritis after 24 h injection^12^. Therefore, targeting local peritoneal macrophages and modulating their phenotype may have potential for alleviating systemic inflammatory diseases.

Anti-inflammatory M2 macrophages have been subdivided into M2a and M2c depending on their functions and gene expression profiles^5^. M2a macrophages are pro-fibrotic and wound-healing cells which produce collagen type VI, fibronectin, and transforming growth factor beta (TGF-β) while M2c macrophages are predominantly responsible for immune regulation which is associated with T-regulatory (Treg) function^13^. M2c macrophages have been reported to be more effective than M2a in suppression of CD4^+^ T-cell infiltration to kidney, therefore more potent than M2a in reduction of renal inflammation and renal injury due to their ability to induce Treg^14^. Therefore, modulation of macrophages to both M2a and M2c phenotypes may provide a synergistic effect in protection against various inflammatory diseases. IL4 and IL10 have been reported to possess anti-inflammatory effect by inducing macrophage polarization to M2a and M2c phenotype, respectively^15^. However, direct administration of the cytokines caused systemic toxicity and disruption of normal immune functions due to their limited half-life which requires repeated and expensive high dosages to achieve therapeutic concentrations^16^. A gene therapy offers a novel approach for *in vivo* polarization of macrophages due to its ability to achieve long-term expression of nucleic acids replacing the frequent administration of the recombinant proteins^3^. Furthermore, nanotechnology combined with gene therapy could improve the safety by localizing the genetic effect at the inflamed areas, hence avoiding systemic action. So far, there has been no report on targeted delivery system for repolarization of macrophages toward an anti-inflammatory M2 phenotype by gene therapy.

In recent years, natural anionic polysaccharide hyaluronic acid (HA)-based nanoparticles have emerged as promising nanocarriers for targeting drugs and genes to tumor cells which over-express CD44, a receptor of HA^17^,^18^,^19^,^20^. HA-based nanogels and HA-modified liposomes have also been used for targeting CD44-overexpressed macrophages^21^,^22^. For gene delivery, HA has been modified with a cationic polymer which has capacity of forming complex with negatively charged nucleic acids^17^. Among various cationic polymers, poly(ethyleneimine) (PEI) has been widely used for nucleic acid delivery due to its very high positive charge which enables efficient electrostatic complexation with negatively charged nucleic acids, and its ability to facilitate endosomal escape via “proton sponge effect” for improved cytosolic delivery^23^. Our group has demonstrated that HA-PEI was effective for specific delivery of siRNA to ovarian and lung cancer cells over-expressing CD44^17^,^24^. However, its potential as a targeted delivery system for plasmid DNA to macrophages has not been investigated yet. In this study, we synthesized HA-PEI conjugate for encapsulation and specific delivery of plasmid DNA expressing IL4 and IL10 genes to macrophages for modulation of their functional polarity toward anti-inflammatory M2a and M2c phenotypes both *in vitro* in J774A.1 macrophages and *in vivo* in peritoneal macrophages of C57BL/6 mice.

## Results

### *In vitro* characterization and uptake of HA-PEI/pDNA nanoparticles in macrophages

**Figure 1:**
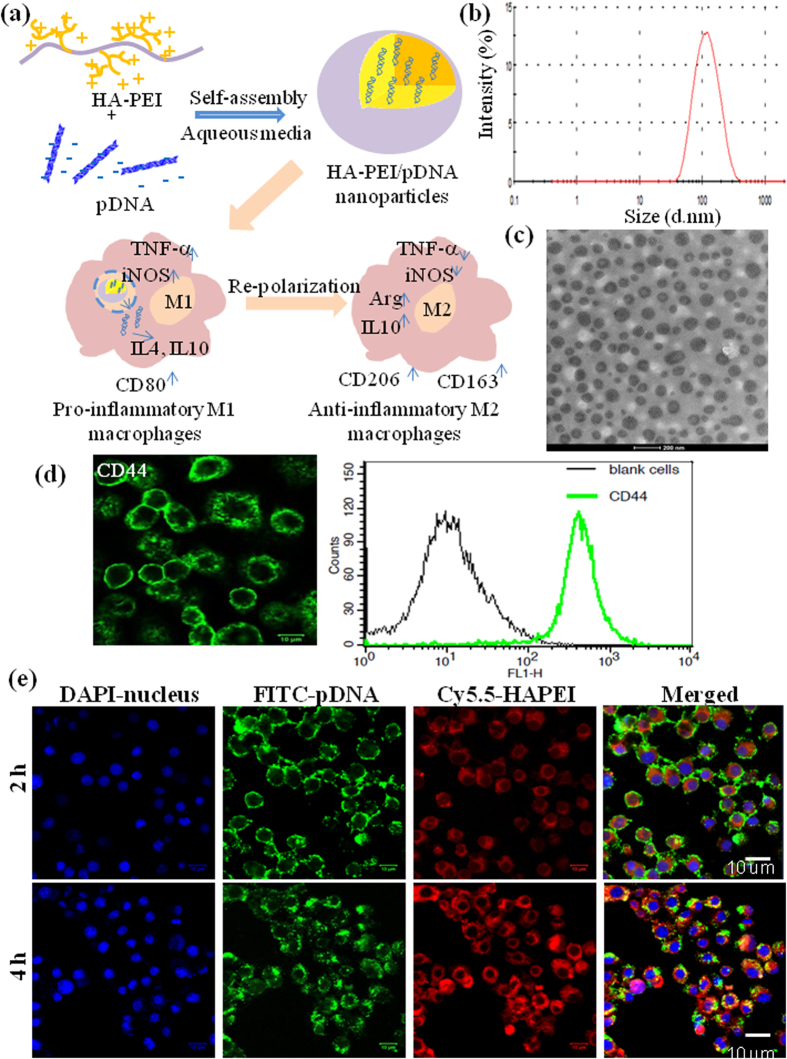
(**a**) Schematic illustration of pDNA encapsulation into HA-PEI nanoparticles for re-polarization of pro-inflammatory M1 macrophages to anti-inflammatory M2 macrophages. (**b**) Size distribution of HA-PEI/pDNA (9:1) in PBS by DLS. (**c**) TEM image of HA-PEI/pDNA in PBS (9:1). (**d**) Confocal microscopy and FACS analysis of CD44 expression in J774A.1 macrophages. (**e**) Uptake of HA-PEI/pDNA nanoparticles in J774A.1 macrophages.

Although PEI is an effective transfecting polymer for nucleic acids, its drawbacks associated with toxicity and rapid clearance by the reticulo-endothelial system limit its application *in vivo*^3^. Therefore, HA was conjugated with PEI to mask the toxicity and render targeting effect of HA for effective transfection of pDNA in macrophages. HA-PEI conjugate was formed via amide bond formation between the carboxyl groups of HA and the amine groups of bPEI using EDC/NHS chemistry. The resulting HA-PEI could form a complex with pDNA via electrostatic interaction between positively charged PEI and negatively charged pDNA (Fig. 1a).

The particle size and morphology of HA-PEI/pDNA NPs (9:1 ratio) were measured by DLS and TEM, respectively. The average particle size of blank HA-PEI NP in PBS was 233.2 nm, which was decreased to 185.9 nm after pDNA encapsulation (Fig. 1b), indicating successful condensation of pDNA into the polymer. TEM showed spherical shape of HA-PEI/pDNA with size in the range of 80–120 nm which was smaller than that measured by DLS (Fig. 1c). HA-PEI/pDNA exhibited negative surface charge in PBS as reflected by the zeta potential value of −11.6 mV, suggesting core/shell structure of the nanoparticles in which the core contained PEI complexed pDNA covered by the hydrophilic shell of negatively charge HA. The core/shell structure of HA-PEI/siRNA was also observed by TEM in our previous study^17^. The HA-PEI/pDNA NPs were stable for at least 72 h at room temperature without visible precipitation.

A pre-requisite for macrophage targeting of HA-based nanoparticles is the high expression of CD44 receptor on the cell membrane. For this purpose, we evaluated CD44 expression on J774A.1 macrophages using confocal microscopy and FACS analysis. Figure 1d shows strong fluorescence on the cell membrane and a significant shift in the histogram of J774A.1 stained with FITC-CD44 antibody compared to that of blank cells, indicating high expression of CD44 on the membrane of J774A.1 cells. We next investigated cellular uptake of HA-PEI/pDNA NPs in J774A.1 macrophages by confocal microscopy. The green signal (FITC) of pDNA and red signal (Cy5.5) of HA-PEI inside the cells increased with increasing incubation time (Fig. 1e). Bright signal of pDNA was observed in the cells after 2 h of incubation, indicating enrichment of pDNA. However, the pDNA mainly located in the cell membrane at 2 h. Increasing the incubation time to 4 h, the strong fluorescence signal of pDNA was diffused through cytoplasm to the nuclear membrane, indicating that HA-PEI/pDNA NPs were effectively internalized by J774A.1 macrophages.

### *In vitro* transfection and polarization study in J774A.1 macrophages

**Figure 2:**
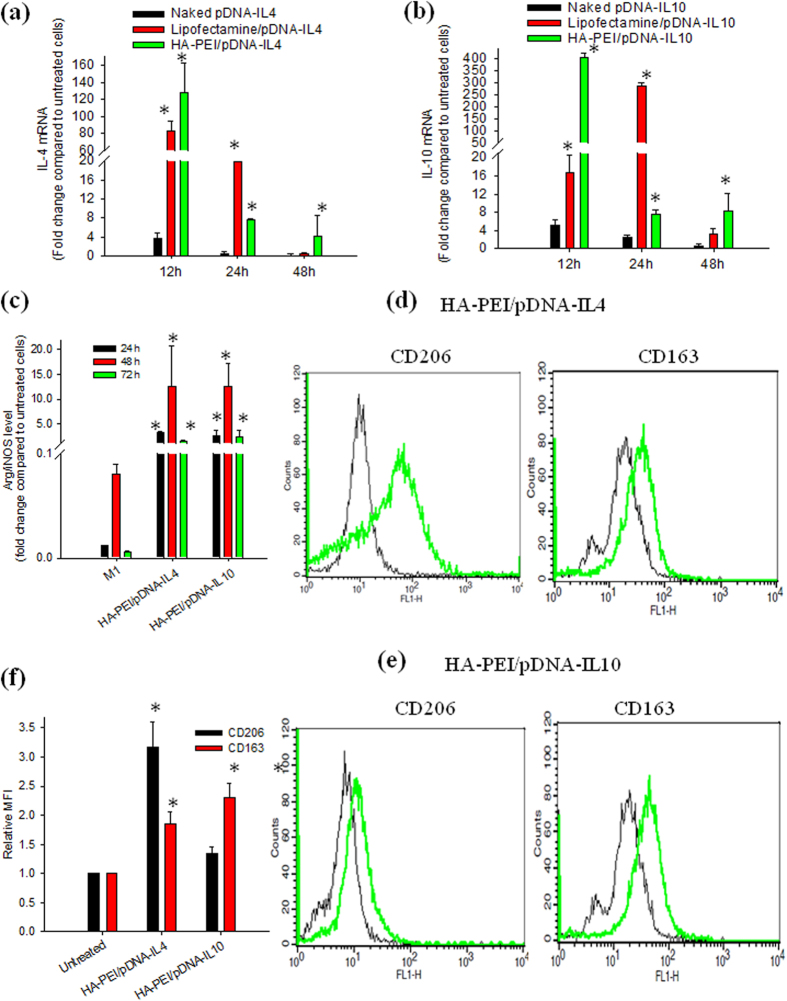
Expression level of (a) IL4 and (b) IL10 measured by qPCR in J774A.1 macrophages transfected with naked plasmid, Lipofectamine®/pDNA, and HA-PEI/pDNA at 12 h, 24 h, and 48 h post-transfection. (**c**) Arg/iNOS (M2/M1) ratio of M1 macrophages transfected with HA-PEI/pDNA-IL4 or HA-PEI/pDNA-IL10 for 24 h, 48 h, and 72 h. Representative flow cytometry histogram of CD206 and CD163 expression in stimulated-J774A.1 macrophages transfected with (**d**) HA-PEI/pDNA-IL4 and (**e**) HA-PEI/pDNA-IL10 for 48 h. (**f**) Relative mean fluorescence intensity (MFI) of CD206 and CD163 expression in macrophages treated HA-PEI/pDNA-IL4, and HA-PEI/pDNA-IL10 for 48 h. n = 3, *p < 0.05 compared to untreated and M1 macrophages.

The ability of HA-PEI NPs to carry and efficiently deliver pDNA-IL4 and pDNA-IL10 was evaluated by *in vitro* transfection of HA-PEI/pDNA NPs in J774A.1 macrophages. qPCR was used for quantitative determination of IL4 and IL10 gene expression levels in J774A.1 macrophages after transfection with HA-PEI/pDNA, naked plasmids, and Lipofectamine®/pDNA at 12 h, 24 h, and 48 h post-transfection (Fig. 2a,b). HA-PEI/pDNA NPs mediated transfection significantly increased IL4 expression up to 130-fold and IL10 expression up to 400 fold after 12 h transfection, indicating fast release and expression of the plasmids from HA-PEI NPs. The expression level of IL4 and IL10 in J774A.1 transfected with Lipofectamine^®^/pDNA was about 80 and 16 fold higher than that in untreated cells at 12 h, respectively. Moreover, transfection levels with HA-PEI/pDNA were even better than those with Lipofectamine^®^/pDNA at 12 h. At 24 h post-transfection, the IL4 and IL10 expression levels were significantly decreased to 8 fold compared to untreated cells in HA-PEI/pDNA transfection while IL10 expression was significantly increased to 25-fold and 250-fold in the transfection with Lipofectamine®/pDNA. However, at 48 h post-transfection, the expression level of IL4 and IL10 in the cells transfected with Lipofectamine® dramatically decreased to similar levels with untreated cells while the IL4 and IL10 levels in the macrophages transfected with HA-PEI/pDNA still remained 5 to 8-fold higher than that in untreated cells. IL10 expression level was significantly higher than IL4 expression level in the macrophages transfected with HA-PEI/pDNA NPs and Lipofectamine®/pDNA. For example, transfection with HA-PEI/pDNA-IL4 induced IL4 expression at 130 fold while HA-PEI/pDNA-IL10 produced higher expression of IL10 at 400 fold compared to untreated cells at 12 h. Likewise, the cells transfected with Lipofectamine®/pDNA had IL4 expression at 20 fold while IL10 expression at 300 fold compared to untreated cells. At all time points, insignificant upregulation of IL4 and IL10 genes occurred when the cells were exposed to naked plasmids. The results indicated that HA-PEI was more effective in maintaining high transgene expression up to 48 h compared to Lipofectamine®, a widely used commercial transfection reagent. Furthermore, it should be noted that Lipofectamine® was very toxic to macrophages; a significant number of cells died after 2 h of incubation with Lipofectamine®/pDNA complex while the cells transfected with HA-PEI/pDNA were healthy throughout the study (data not shown), indicating that HA-PEI was a suitable carrier for plasmid DNA delivery to macrophages.

To establish a system for macrophage polarization studies, J774A.1 macrophages were stimulated with different stimuli, LPS and IFN-γ or IL4 cytokine, and the expression level of M1 and M2 markers were determined by PCR. Stimulation of J774A.1 macrophages with LPS and IFN-γ for 16 h induced M1 macrophages with high level of iNOS, TNF-α, and CD80 receptor while the expression of Arg and IL10 was similar to that of untreated cells (Figure S2A,B). In contrast, J774A.1 stimulated with IL4 or IL10 cytokines for 16 h upregulated the expression of Arg and IL10 with low expression of iNOS and TNF-α (Figure S2A). Furthermore, macrophages stimulated with IL4 significantly increased expression of CD206 receptor and CD163 expression, surface markers for M2 macrophages^13^,^25^ (Figure S2C). However, treatment with IL10 increased CD163 expression on the macrophage surfaces while insignificant change in the CD206 expression was observed (Figure S2D). The results suggest that both CD206 and CD163 was specific surface marker for M2a macrophages while only CD163 specifically expressed in M2c phenotype. Since polarization of macrophages toward M2 phenotype was associated with a decrease in iNOS expression level and an increase in Arg level, a ratio of Arg/iNOS was selected as an indication of M2 macrophage polarization.

To investigate if high expression of IL4 and IL10 gene in the macrophages could re-polarize the macrophages from M1 to M2a and M2c phenotypes, qPCR was used to measure Arg/iNOS ratio in the M1 phenotype transfected with HA-PEI/pDNA-IL4 and HA-PEI/pDNA-IL10 NPs for 24 h, 48 h, and 72 h (Fig. 2c). Due to high expression of iNOS and low expression of Arg in M1 macrophages, the Arg/iNOS ratio was low in the M1 cells. However, this ratio was significantly increased in M1 macrophages transfected with HA-PEI/pDNA-IL4 and HA-PEI/pDNA-IL10 NPs in which the highest value was achieved at 48 h post-transfection, which was 10^5^ fold higher than that in the M1 macrophages. The results indicated that J774A.1 macrophages were effectively re-polarized from M1 to M2 state by pDNA-IL4 and pDNA-IL10 encapsulated in HA-PEI NPs. Furthermore, the polarizing effect occurred at 24 h and maintained up to 72 h, possibly due to the sustained effect by pDNA. In addition, the increased Arg/iNOS ratio by IL4 and IL10 gene was comparable at all time points, indicating similar polarizing effect by HA-PEI/pDNA-IL4 and HA-PEI/pDNA-IL10 NPs.

To confirm the macrophage polarizing effect by HA-PEI/pDNA-IL4 and HA-PEI/pDNA-IL10 NPs, we evaluated the change in the expression of CD206 and CD163 in J774A.1 macrophages treated with HA-PEI/pDNA-IL4 and HA-PEI/pDNA-IL10 NPs by FACS analysis. Figure 2d–f show histograms and mean fluorescence intensity of CD206 and CD163 expression in M1 macrophages transfected with HA-PEI/pDNA-IL4 and HA-PEI/pDNA-IL10 for 48 h. Compared to untreated macrophages, there was a shift in the histogram and significant increase in the fluorescence intensity of CD206 and CD163 in the cells treated with HA-PEI/pDNA NPs, indicating upregulation of M2 surface markers CD206 and CD163 in macrophages treated with HA-PEI/pDNA-IL4. However, fluorescence intensity of only CD163 was increased in the cells polarized with HA-PEI/pDNA-IL10. In addition, the mean fluorescence intensity of M2 macrophages polarized with HA-PEI/pDNA-IL4 and HA-PEI/pDNA-IL10 was comparable to those in M2a and M2c induced by IL4 and IL10 cytokines, respectively. Furthermore, expression of CD80 in M1 macrophages was decreased when the macrophages were transfected with HA-PEI/pDNA-IL4 and HA-PEI/pDNA-IL10 (Figure S3B,C). The upregulation of Arg/iNOS and M2 surface markers indicated successful polarization of J774A.1 macrophages toward anti-inflammatory M2 phenotypes by pDNA-IL4 and pDNA-IL10-encapsulated HA-PEI NPs.

### *In vivo* uptake and polarization study in peritoneal macrophages of C57BL/6 mice

**Figure 3:**
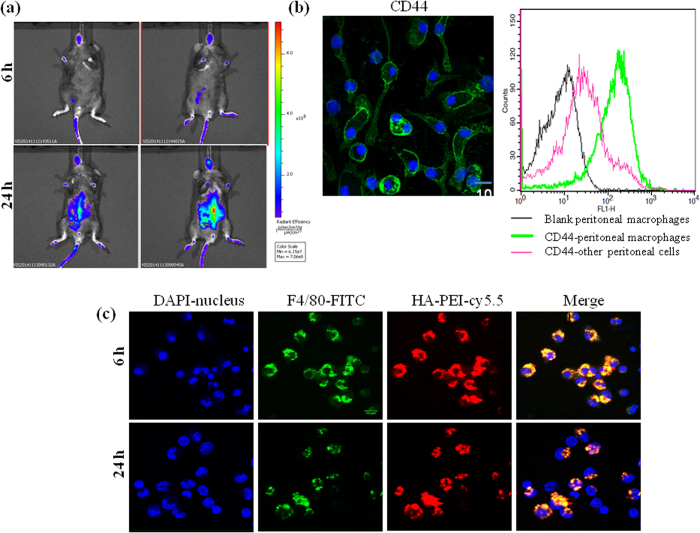
(**a**) Whole body near-IR fluorescence imaging of Cy5.5-conjugated HA-PEI nanoparticles upon IP administration in C57BL/6 mice. (**b**) CD44 expression in peritoneal macrophages by confocal microscopy and FACS analysis. (**c**) *In vivo* uptake of Cy5.5 labeled HA-PEI nanoparticles in peritoneal macrophages via IP administration. The cells were extracted from C57BL/6 mice at 6 h and 24 h post-injection for staining with macrophage specific F4/80 antibody. The co-localization of red signal from HA-PEI nanoparticles and green signal of F4/80 antibody indicated the uptake of HA-PEI in peritoneal macrophages.

To evaluate if HA-PEI NPs can target peritoneal macrophages *in vivo* following IP administration, Cy5.5-conjugated HA-PEI dispersed in PBS was injected to peritoneal cavity of C57Bl/6 mice which were pre-injected intraperitoneally with thioglycollate. *In vivo* imaging showed low signal of HA-PEI NPs at 6 h post-IP administration, which might be due to the distribution of the NPs throughout the peritoneal cavity without accumulated region (Fig. 3a). At 24 h post-injection, strong fluorescence signal was observed in the abdominal of the mice which were correlated with the nanoparticle accumulation in liver, kidney, and spleen of the mice (Figure S4). At 6 h and 24 h post-injection of the nanoparticles, peritoneal macrophages were extracted and stained with anti-mouse F4/80 antibody which is macrophage specific antibody^26^. We also evaluated CD44 expression on peritoneal macrophages using confocal microscopy and FACS analysis. Figure 3b shows strong fluorescence on the cell membrane and a significant shift in the histogram of peritoneal macrophages stained with FITC-CD44 antibody compared to that of blank peritoneal macrophages and other peritoneal cells, indicating high expression of CD44 on the membrane of peritoneal macrophages. Confocal microscopy showed strong green signal of F4/80 and red signal of HAPEI in the cytoplasm at both 6 h and 24 h post-injection (Fig. 3c). Importantly, these signals were co-localized, indicating that HA-PEI NPs effectively targeted peritoneal macrophage over-expressing CD44 following IP administration.

**Figure 4:**
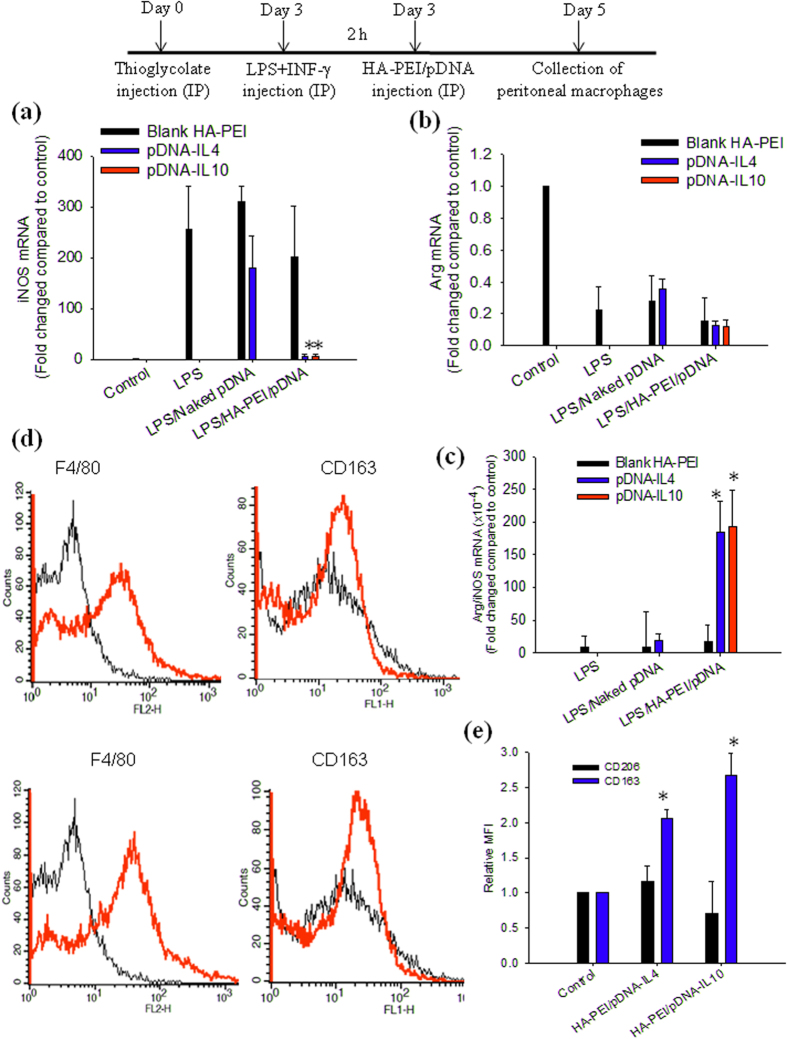
*In vivo* polarization study in peritoneal macrophages. Expression of (**a**) iNOS mRNA, (**b**) Arg mRNA, and (**c**) Arg/iNOS ratio (M2/M1) in LPS and IFN-γ-stimulated peritoneal macrophages treated with naked plasmids (IL4 and IL10), and HA-PEI/pDNA (IL4 and IL10). qPCR was used for quantification of gene expression level (n = 4). *p < 0.05 compared to the group treated with LPS and IFN-γ. (**d**) FACS analysis of *in vivo* polarized peritoneal macrophages. Representative flow cytometry histogram of F4/80 and CD163 expression in peritoneal macrophages treated with HA-PEI/pDNA-IL4 and HA-PEI/pDNA-IL10; (**e**) Relative mean fluorescence intensity (MFI) of CD206 and CD163 expression in *in vivo* polarized peritoneal macrophages compared to un-polarized peritoneal macrophages. The peritoneal macrophages were extracted from C57BL/6 mice at 48 h post-IP administration of plasmid DNA. *p < 0.05 compared to control group, n = 4.

To investigate the potential of HA-PEI/pDNA-IL4 and HA-PEI/pDNA-IL10 NPs for modulation of peritoneal macrophage functional polarity *in vivo*, an *in vivo* model of M1 peritoneal macrophages was induced by IP injection of LPS and IFN-γ in C57BL/6 mice which were pre-injected intraperitoneally with thioglycollate for peritoneal macrophage recruitment. The M1 peritoneal macrophages were then treated with naked pDNA, HA-PEI/pDNA-IL4 or HA-PEI/pDNA-IL10 NPs via IP administration. Figure 4a,b shows expression levels of iNOS and Arg in peritoneal macrophages extracted from C57BL/6 mice at 48 h post-treatment. Peritoneal macrophages were successfully polarized to M1 phenotype *in vivo* upon stimulation with LPS and IFN-γ as indicated by an increase in the iNOS level up to 250 fold compared to the control group. Treatment with naked plasmids insignificantly changed iNOS level in M1 macrophages indicating negligible effect of the plasmids, possibly due to the plasmid degradation inside the mouse peritoneal cavity and inefficient uptake of free plasmid in peritoneal macrophages. Treatment with HA-PEI/pDNA-IL4, and HA-PEI/pDNA-IL10 NPs significantly downregulated iNOS expression level as evident by a decrease iNOS level from 255 fold to about 5 fold compared to the control group (Fig. 4a). The results indicated that HA-PEI/pDNA-IL4 and HA-PEI/pDNA-IL10 efficiently decreased inflammatory M1 macrophage population *in vivo*, in which HA-PEI/pDNA-IL4 and HA-PEI/pDNA-IL10 has similar effect. Stimulation with LPS and IFN-γ decreased Arg expression level in M1 peritoneal macrophages to 80% compared to control group. However, the Arg level in the macrophages did not increase after treatment with HA-PEI/pDNA (Fig. 4b). Due to the significant decrease in iNOS level, the Arg/iNOS ratio (M2/M1) significantly increased in the macrophages treated with HA-PEI/pDNA compared to that in M1 macrophages (Fig. 4c), indicating that peritoneal macrophage spectrum shifted from M1 to M2 phenotype *in vivo* upon treatment with HA-PEI/pDNA-IL4 and HA-PEI/pDNA-IL10.

To investigate the influence of HA-PEI/pDNA-IL4 and HA-PEI/pDNA-IL10 NPs on CD206 and CD163 expression level in peritoneal macrophages *in vivo*, peritoneal macrophages treated with HA-PEI/pDNA-IL4 and HA-PEI/pDNA-IL10 NPs extracted from the mice at 48 h post-treatment were double stained with anti-mouse F4/80 and CD206 or CD163 antibodies for FACS analysis (Fig. 4d). A significant shift in the histogram of the cells stained with F4/80 antibody confirmed the macrophages used for the analysis. Furthermore, a shift in the histogram of the macrophages stained with CD163 along with more than 2 fold-increases in the mean fluorescence intensity of the cells treated with both HA-PEI/pDNA-IL4 and HA-PEI/pDNA-IL10 NPs indicated the upregulation of this M2 surface marker (Fig. 4d–e). However, CD206 expression was insignificantly changed by the treatment (Fig. 4e). The results indicated that *in vivo* polarized peritoneal macrophages behaved differently compared to *in vitro* polarized J774A.1 macrophages in which Arg and CD206 levels were upregulated *in vitro* but not *in vivo*.

### *In vivo* anti-inflammatory effect of HA-PEI/pDNA NPs in C57BL/6 mice

**Figure 5:**
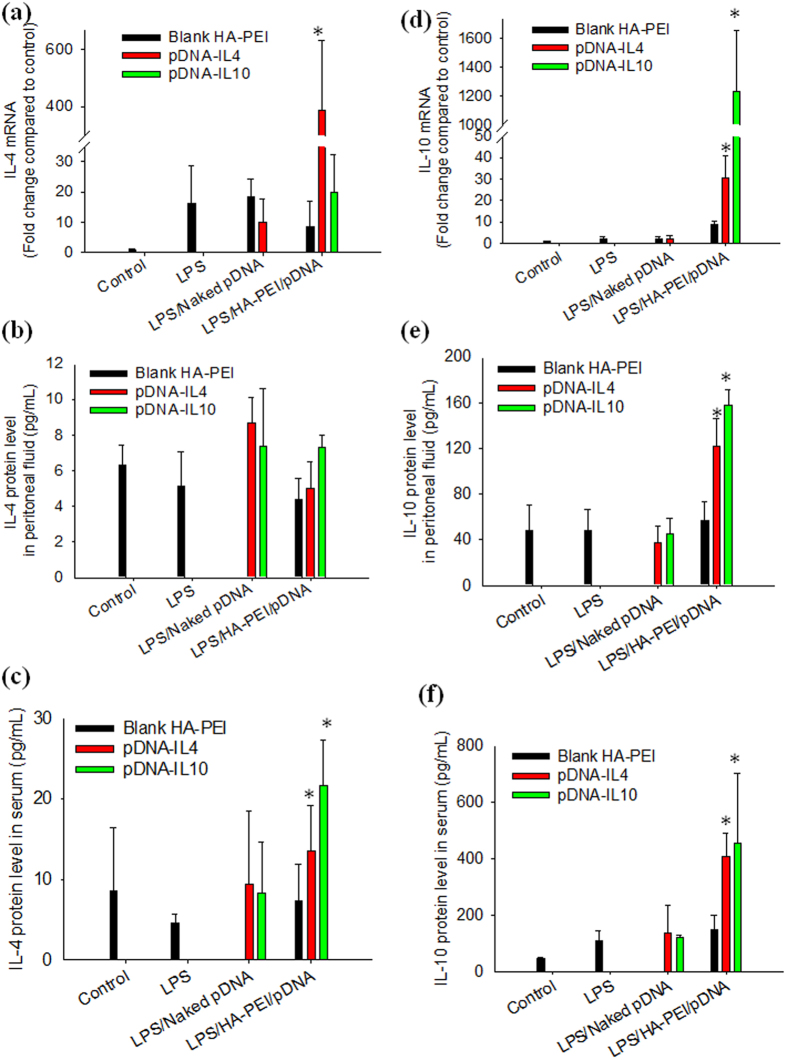
*In vivo* anti-inflammatory study: expression of anti-inflammatory cytokines IL4 and IL10 at both transcript and protein levels at 48 h post-nanoparticle injection. mRNA expression (**a**) IL4 and (**d**) IL10. Peritoneal cytokine level (**b**) IL4 and (**e**) IL10. Serum cytokine level (**c**) IL4 and (**f**) IL10. qPCR and ELISA were used to quantify mRNA and protein levels, respectively. *p < 0.05 compared to LPS-treated group, n = 3.

Following the switch of peritoneal macrophages to the M2 phenotype, we investigated *in vivo* anti-inflammatory effect of HA-PEI/pDNA NPs in a LPS-induced inflammation model in C57BL/6 mice. The mice were injected intraperitoneally with HA-PEI/pDNA NPs 24 h before IP administration of LPS at a higher dose used in the polarization study. Figure 5a–f displays IL4 and IL10 expression level at 48 h post-IP administration. LPS treatment did not significantly change IL4 and IL10 expression at both mRNA and protein levels compared to the control group. *In vivo* transfection of peritoneal macrophages with HA-PEI/pDNA increased expression level of IL4 mRNA to 400 fold and IL10 mRNA to 1200 fold compared to the control group (Fig. 5a,d), indicating that HA-PEI/pDNA NPs efficiently transfected the genes to the cells *in vivo* in which IL10 transgene expression was markedly higher than IL4 expression. At protein level, treatment with both HA-PEI/pDNA-IL4 and HA-PEI/pDNA-IL10 did not change peritoneal IL4 level while increasing serum IL4 level (12 pg/mL in HA-PEI/pDNA-IL4 group and 20 pg/mL in HA-PEI/pDNA-IL10 group) compared to LPS treatment group (5 pg/mL) (Fig. 5b,c). IL10 cytokine level was significantly increased in both peritoneal fluid and serum in the groups treated with both HA-PEI/pDNA-IL4 and HA-PEI/pDNA-IL10 in which serum IL10 level (400 pg/mL in HA-PEI/pDNA-IL4 and 410 pg/mL in HA-PEI/pDNA-IL10 treatment group) was markedly higher than that in peritoneal fluid (120 pg/mL in HA-PEI/pDNA-IL4 and 160 pg/mL in HA-PEI/pDNA-IL10 treatment group) (Fig. 5e,f). Treatment with naked pDNA and blank HA-PEI NPs insignificantly changed IL4 and IL10 expression at both mRNA and protein levels compared to LPS treatment group (Fig. 5a–f). The results indicated that IP administration of HA-PEI/pDNA-IL4 and HA-PEI/pDNA-IL10 increased both local and systemic IL10 cytokine while only systemic IL4 level was increased at significantly lower level than the IL10 levels.

**Figure 6:**
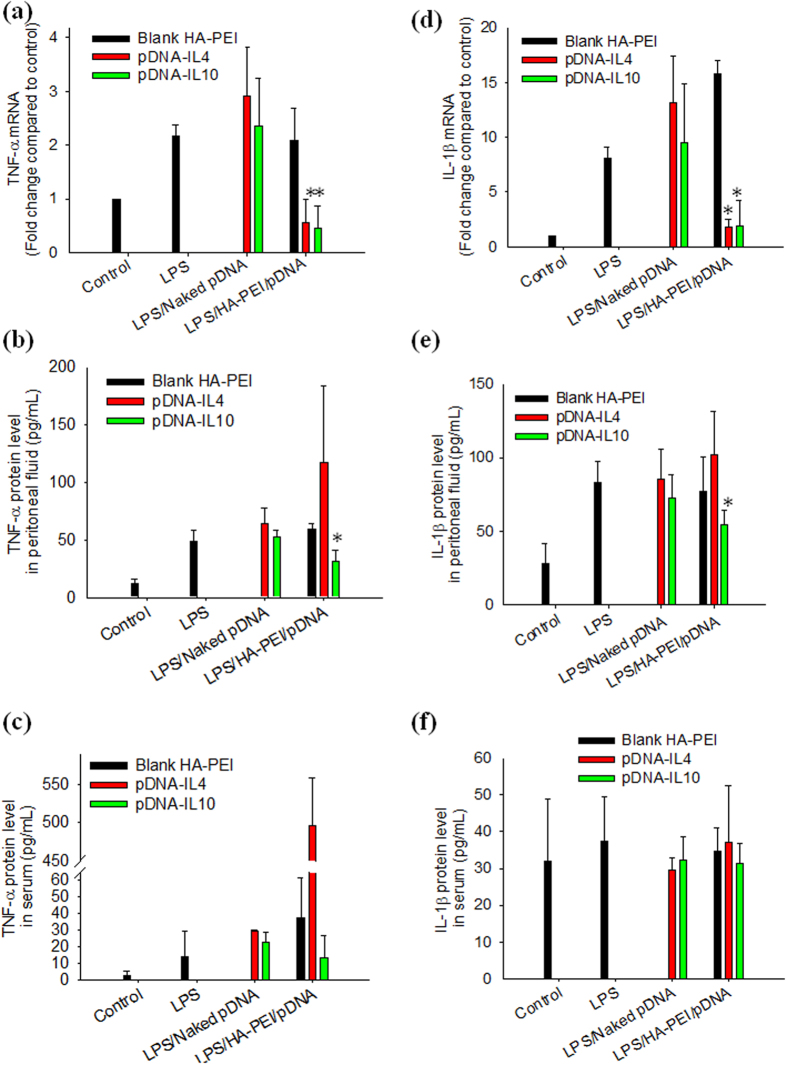
*In vivo* anti-inflammatory study: expression of pro-inflammatory cytokines TNF-α and IL-1β at both transcript and protein level at 48 h post-nanoparticle injection. mRNA expression (**a**) TNF-α and (**d**) IL-1β. Peritoneal cytokine level (**b**) TNF-α and (**e**) IL-1β. Serum cytokine level (**c**) TNF-α and (**f**) IL-1β. qPCR and ELISA were used to quantify mRNA and protein levels, respectively. *p < 0.05 compared to LPS-treated group, n = 3.

Treatment with LPS for 24 h upregulated TNF-α mRNA and IL-1β mRNA in peritoneal macrophages to 2.3 fold and 7.5 fold, respectively, compared to control group (Fig. 6a,d). Similarly, protein levels of TNF-α and IL-1β in peritoneal fluid were significantly increased compared to control group upon LPS stimulation (50 pg/mL vs 10 pg/mL for TNF-α and 80 pg/mL vs 25 pg/mL for IL-1β) (Fig. 6b,e). However, level of TNF-α and IL-1β in serum insignificantly changed in the LPS treated group compared to control group (Fig. 6c,f), indicating that LPS treatment caused local inflammation in the peritoneal cavity but not systemic inflammation. Treatment with naked pDNA and blank HA-PEI NPs did not change the level of TNF-α and IL-1β in both peritoneal fluid and serum compared to LPS treatment group. However, when the mice were treated with HA-PEI/pDNA-IL10, TNF-α and IL-1β levels was significantly decreased to 25 pg/mL and 50 pg/mL, respectively (Fig. 6b,e) which was in accordance with qPCR data, indicating that HA-PEI/pDNA-IL10 suppressed the inflammation caused by LPS. Unexpectedly, TNF-α level in both peritoneal fluid and serum was significantly increased in HA-PEI/pDNA-IL4 treatment group while the treatment did not affect IL-1β level (Fig. 6b–f), despite HA-PEI/pDNA-IL4 significantly increased both serum and peritoneal IL10 levels. The reason for increasing TNF-α protein level by HA-PEI/pDNA-IL4 is currently unknown. The results indicated that HA-PEI/pDNA-IL10 was more effective than HA-PEI/pDNA-IL4 in suppressing LPS-induced inflammation.

## Discussion

Macrophages play a central role in the pathogenesis of various inflammatory diseases, thereby representing an important target for immunotherapy. However, transfection and release of nucleic acids in macrophages are very challenging, especially in primary macrophages due to their highly degradative phagocytic, endosomal, and lysosomal compartments^27^,^28^, which requires an effective vehicle for delivery of pDNA to macrophages. In this study, HA-PEI was selected in this study based on the high capacity of PEI for encapsulation of nucleic acids and targeting ability of HA to CD44-overexpressed cells^17^. Our FACS and confocal microscopy results showed high expression of CD44 on the membrane of J774A.1 macrophages and peritoneal macrophages. The high expression of CD44 on the membrane of both established macrophage cell line and primary peritoneal macrophages confirmed the potential target of these cells for HA-based nanoparticles. *In vitro* cellular uptake study showed efficient uptake of HA-PEI/pDNA NPs to J774A.1 macrophages, possibly via CD44 receptor recognition which might promote the interaction of the cell membrane and the NPs, leading to enhanced endocytosis^29^. The effective internalization of HA-PEI/pDNA then produced high expression of IL4 and IL10 gene in the macrophages in a sustained manner, indicating that HA-PEI was an effective delivery system for targeting pDNA to macrophages.

The highly plastic macrophages can switch their polarity to M1 or M2 phenotype depending on the presence of soluble factors in the local environment^30^. The two phenotypes of macrophages show characteristic expression profiles of surface markers as well as cytokines and chemokines. In addition, M2 macrophages exhibit an elongated shape compared to M1 macrophages^31^. We demonstrated that J774A.1 macrophages stimulated with LPS and IFN-α polarized to M1 phenotype with high expression of iNOS, TNF-α, and CD80. In contrast, stimulation of J774A.1 with IL4 or IL10 cytokine induced M2 macrophages characterized by high expression of Arg, IL10, CD206 and CD163 in which CD163 was more specific for IL10-stimulated macrophages while IL4-stimulation upregulated both CD206 and CD163 expression. Accumulating evidence has shown that polarization of macrophages toward M2 phenotype was due to stimulation of signal transducer and activator of transcription 6 (STAT-6) by IL4 and STAT-3 activation by IL10^32^. Despite high potency for M2 macrophage polarization, the use of IL4 and IL10 cytokines is limited due to their toxicity. To overcome this drawback, we employed gene therapy with pDNA as an alternative approach. We found that transfection of stimulated J774A.1 macrophages with HA-PEI/pDNA-IL4 and HA-PEI/pDNA-IL10 could re-polarize the M1 macrophages to M2 macrophages. This was evident by a significant increase in Arg/iNOS up to 72 h post-transfection together with induction of CD206 and CD163 receptors. Furthermore, upregulation of M2 surface markers by HA-PEI/pDNA-IL4 and HA-PEI/pDNA-IL10 was comparable to those by IL4 and IL10 cytokines. The results indicated successful modulation of J774A.1 macrophages toward an anti-inflammatory phenotype. However, the successful M2 macrophage polarization in an *in vitro* culture model system may not be comparable with an *in vivo* situation.

We next established an *in vivo* model of M1 peritoneal macrophages and administered HA-PEI/pDNA via IP injection. It has been demonstrated that upregulation of inflammatory cytokines such as IL-1, IL-6, and TNF-α in peritoneal macrophages occurred during the acute and chronic phase of experimental rheumatoid arthritis^33^, suggesting that these cells involved in systemic immunity. Likewise, reduced inflammation shown in an arthritic rat model by depleting peritoneal macrophages via IP administration of chlodronate-loaded liposome confirmed potential therapeutic target of these macrophages^34^. Our approach is to directly inject nanoparticles into peritoneal cavity to allow gene delivery into serum-free environment rich in peritoneal macrophages involved systemic immunity. In addition, interactions of the nanoparticles with blood components that potentially induce immune responses or stability issues could be avoided^35^. HA-PEI NPs were found in peritoneal macrophages at 6 h post-administration, indicating effective targeting of the NPs to these cells among various cell types in the mouse peritoneal cavity. Furthermore, we found that *in vivo* transfection of HA-PEI/pDNA-IL4 and HA-PEI/pDNA-IL10 to peritoneal macrophages shifted the macrophages from M1 to M2 phenotype in which iNOS level was significantly reduced without affecting level of Arg expressed despite the presence of high IL-4 and IL-10 gene levels in the cells. This might be due to high level of Arg in thioglycolate-elicited peritoneal macrophages, leading to the difficulty in modulating Arg level. Similar trend has also been reported by Courties *et al*. in which *in vivo* silencing the transcript factor IRF5, an M1 activator, by siRNA decreased M1 macrophage-associated genes without increasing M2 genes. The reduced expression of inflammatory M1 macrophage markers by silencing IRF5 supported resolution of inflammation, accelerated cutaneous and infarct healing^36^.

We found that *in vitro* transfection of HA-PEI/pDNA in J774A.1 macrophages increased M2 surface markers in which HA-PEI/pDNA-IL4 increased both CD206 and CD163 expression while HA-PEI/pDNA-IL10 only increased CD163 expression. However, *in vivo* polarization of peritoneal macrophages with both HA-PEI/pDNA-IL4 and HA-PEI/pDNA-IL10 increased CD163 expression without changing expression of CD206. The difference in the expression of macrophage surface markers upon polarization with HA-PEI/pDNA-IL4 and HA-PEI/pDNA-IL10 in J774A.1 macrophages and in peritoneal macrophages might be due to difference in the macrophage behavior *in vitro* and *in vivo*. Mouse macrophages from different sites have been reported to expression different levels of surface markers in which peritoneal macrophages have low expression of CD206 while alveolar macrophages have high expression of this receptor^37^. This low CD206 expression was not changed by the treatment with HA-PEI/pDNA. Previous studies have shown that peritoneal M1 macrophages could be polarized to M2 phenotype *in vitro* by stimulation with IL4 cytokine as indicated by the reduction in TNF-α level and induction of CD206 expression. However, IP administration of IL4 cytokine failed to reverse peritoneal M1 macrophages to M2 *in vivo* in which no change in the TNF-α level and CD206 expression was observed in the activated peritoneal macrophages after 2 h treatment with IL4 cytokine^38^. This might be due to the short treatment time and fast degradation of IL4 cytokine in the ascitic fluid upon IP administration^38^. In our study, we found that hyaluronic acid nanoparticle-based gene delivery system was able to target peritoneal macrophages and modulated their polarity from M1 to M2 phenotype *in vivo* as evident by the decrease of iNOS and increase of CD163 expression.

Following the macrophage phenotype switch, we investigated *in vivo* anti-inflammatory effect of HA-PEI/pDNA NPs in a LPS-induced inflammation model. We found that IP administration of HA-PEI/pDNA-IL10 significantly increased peritoneal and serum levels of IL10 but not IL4 which reduced peritoneal TNF-α and IL-1β levels, indicating that the local inflammation caused by LPS was effectively suppressed by HA-PEI/pDNA-IL10. Therefore, modulation of peritoneal macrophage toward the anti-inflammatory phenotype *in vivo* may be beneficial in decreasing various inflammatory diseases by reducing a range of inflammatory mediators. However, treatment with HA-PEI/pDNA-IL10 did not influence serum TNF-α and IL-1β levels due to insignificant increase of these cytokines in serum at 24 h post IP injection of LPS compared to control group. The lack of effect of LPS on serum TNF-α and IL-1β levels might be due to the increase in serum levels of these cytokines by LPS at earlier time points.

In conclusion, we demonstrated for the first time the polarization of macrophages toward anti-inflammatory phenotypes both *in vitro* and *in vivo* by plasmid DNA expressing IL4 and IL10 encapsulated in CD44 targeting nanoparticles (HA-PEI/pDNA). HA-PEI/pDNA self-assembled to form nanoparticles which were efficiently internalized by J774A.1 macrophages over-expressing CD44 receptor. Importantly, *in vitro* transfection of the macrophages with HA-PEI/pDNA-IL4 and HA-PEI/pDNA-IL10 produced high level of IL4 and IL10 genes in the macrophages which could increase Arg1/iNOS2 gene expression levels and up-regulate CD206 and CD163 expression. Upon IP administration in C57BL/6 mice, CD44 targeting hyaluronic acid nanoparticle-based gene delivery system was able to target peritoneal macrophages and modulate their functional polarity. *In vivo* transfection of HA-PEI/pDNA-IL4 and HA-PEI/pDNA-IL10 in stimulated peritoneal macrophages upregulated IL4 and IL10 genes and increased peritoneal and serum IL10 levels which switched LPS and IFN-γ stimulated peritoneal macrophage toward M2 phenotype. Furthermore, IP administration of HA-PEI/pDNA-IL10 NPs effectively suppressed local inflammation induced by LPS. The results indicated that peritoneal macrophages were successfully polarized from M1 to M2 phenotype by HA-PEI/pDNA-IL4 and HA-PEI/pDNA-IL10 NPs *in vivo* which may have potential for the treatment of various inflammatory diseases.

## Experimental Methods

### Materials

Sodium hyaluronate (HA, 20,000 Da) was purchased from Lifecore Biomedical Co. (Chaska, MN). Branched poly(ethyleneimine) (bPEI, 10,000 Da) was obtained from Polysciences Inc. (Warrington, PA). N-(3-dimethylaminopropyl)-N’-ethylcarbodiimide hydrochloride (EDC), N-hydroxysuccinimide (NHS), Brewer-thioglycollate medium, and lipopolysaccharide (LPS) were purchased from Sigma-Aldrich Chemical Co. (St. Louis, MO, USA). J774A.1 adherent murine macrophage cell line obtained from the American Type Culture Collection (ATCC; Manassas, VA) was cultured in Dulbecco’s modified Eagle medium (DMEM) (Cellgro, Manassas, VA) containing 10% fetal bovine serum (FBS) (HyClone, Logan, UT), and penicillin/streptomycin antibiotics (Gibco Invitrogen, Woburn, MA) at 37 °C and 5% CO_2_. Rabbit anti-CD163/M130 polyclonal antibody conjugated with Alexa Fluor 488 dye was purchased from Bioss Antibodies (Woburn, MA). Alexa Fluor 488-anti-mouse CD44, CD80 and CD206 antibodies were obtained from BioLegend (San Diego, CA). Recombinant murine interleukin 4 (IL4), interleukin 10 (IL10), and interferon-gamma (IFN-γ) were obtained from PeproTech (Rocky Hill, NJ). *E. coli* GT116 cells, pUNO1-mIL4 and pUNO1-mIL10 plasmids, blasticidin, and 4’, 6-diamidino-2-phenylindole (DAPI) were purchased from Invivogen (San Diego, CA). Plasmid Mega Kits were obtained from Qiagen (Valencia, CA). 1% EX E-gels, nuclease-free water, and Lipofectamine® 2000 were purchased from Life Technologies (Woburn, MA). The *Label* IT Intracellular Localization Kit was received from Mirus (Madison, WA). Primers specific for iNOS-2, TNF-α, Arg-1, IL-4, IL-10 and β-actin were purchased from Eurofins MWG Operon (Huntsville, AL).

### Amplification of plasmid DNA

To amplify plasmid DNA, competent *E. coli* GT116 cells were transformed with pUNO1-mIL4 and pUNO1-mIL10 plasmids, containing murine IL4 and IL10 genes respectively, through a heat- shock method. Briefly, 10 *μ*L of plasmid DNA was added to 0.5 mL of reconstituted bacterial cells and incubated on ice for 30 min. The bacterial solution was subsequently heated in a water bath at 42 °C for 30 sec and then returned to ice for 2 min. The bacterial culture was then allowed to grow for 2 h in a shaker at 37 °C. To select bacteria containing IL4 or IL10 plasmids, bacteria were cultured on LB (Luria broth) agar plates and in LB medium containing blasticidin. Because the plasmids contained an insert *Bsr* gene conferring blasticidin resistance, only bacteria containing the plasmids and thus the IL4 and IL10 genes amplified in blasticidin-containing medium. Plasmids were then harvested and purified using a Plasmid Mega Kit following the manufacturer’s instructions. The plasmid purity was confirmed by measuring the absorbance ratio at 260/280 nm and agarose gel electrophoresis. The presence of IL4 and IL10 genes within the plasmids was confirmed by double digesting the plasmids at restriction sites surrounding the two genes, and running on a 1% EX E-gel.

### Synthesis of HA-PEI conjugate, preparation and characterization of HA-PEI/pDNA NPs

HA-PEI conjugate was synthesized by coupling reaction between HA and PEI in the presence of EDC/NHS. Briefly, HA (100 mg) and PEI (15 mg) were dissolved in distilled water (10 mL) containing NaCl (0.5 M). A solution containing EDC (35 mg) and NHS (22 mg) dissolved in distilled water was then added to the polymer mixture. The reaction mixture was stirred at room temperature for 24 h and then dialyzed against distilled water using a dialysis membrane (MWCO: 25 kDa) for 24 h. The HA-PEI conjugate was obtained after lyophilization. The lyophilized HA-PEI conjugate dissolved in D_2_O was characterized by 500 MHz ^1^H NMR spectroscopy (Varian Inc., CA).

HA-PEI/pDNA-IL4 and HA-PEI/pDNA-IL10 nanoparticles (NPs) were prepared by mixing the HA-PEI solution in phosphate buffer saline (PBS, pH 7.4) with pDNA at an appropriate ratio (w/w) using a vortex mixer and incubating the complex for 10 min at room temperature.

The average particle size, size distribution and zeta-potential of HA-PEI/pDNA NPs were measured using a dynamic light scattering (DLS) instrument (Malvern Zetasizer, Westborough, MA). The morphology of the NPs was imaged by Transmission Electron Microscopy (TEM) (JEOL, Peabody, MA). Specimens were prepared by adding a suspension of the nanoparticles dropwise to a Formvar/carbon film grid followed by air drying.

To determine the optimal ratio of HA-PEI to plasmid DNA for the formation of HA-PEI/pDNA NPs, different NPs were prepared with different HA-PEI to pDNA ratios (w/w): 54:1, 27:1, 9:1, and 5:1. The nanoparticles were then run on 1% EX E-gel to check percent encapsulation of the plasmid. Plasmid encapsulation was further confirmed by decomplexing HA-PEI/pDNA-IL4 and HA-PEI/pDNA-IL10 with anionic polyacrylic acid (PAA) by mixing an equal volume of HA-PEI-pDNA and PAA using a vortex mixer. The strongly anionic PAA displaces the plasmid by electrostatically interacting with the cationic PEI. The decomplexed samples were then run on a 1% EX E-gel to ensure the presence of intact plasmid bands.

### Intracellular uptake of HA-PEI/pDNA in J774A.1 macrophages

To observe the cellular uptake, the pDNA was previously labeled with a green fluorescence dye (FITC) using a *Label* IT Intracellular Localization Kit and HA-PEI was also pre-labeled with a red fluorescence dye, Cy5.5. J774A.1 macrophages were seeded at a density of 1.0 × 10^5^ cells/well in an 8-well chamber of a Lab-Tek II chamber slide and pre-incubated for 24 h at 37 °C and 5% CO_2_. Serum-free DMEM containing HA-PEI/pDNA NPs at an equivalent dose of 4 *μ*g of DNA was added to each well, followed by incubation for 2 h and 4 h at 37 °C. After incubation, the cells were washed twice with PBS and fixed in formalin (4%) for 15 min at room temperature, followed by nucleus staining with 1 μg/mL of DAPI. Cover glasses were then placed on glass slides. The cellular uptake of HA-PEI/pDNA NPs was imaged on a Zeiss confocal microscope (Carl Zeiss, Cambridge, UK) at an excitation wavelength of 488 nm for pDNA and 647 nm for Cy5.5.

### Analysis of CD44, CD80, CD206 and CD163 expression on macrophage by flow cytometry

J774A.1 macrophages at 80% confluency were plated in T25 flasks (400,000 cells per flask) and cultured at 37 °C and 5% CO_2_. The cells were polarized to M1 phenotype by incubating with LPS (100 ng/mL) and IFN-γ (100 ng/mL) for 6 h. The M1 macrophages were then transfected with HA-PEI/pDNA-IL4 and HA-PEI/pDNA-IL10 NPs at 40 μg equivalent dose of pDNA-IL4 or pDNA-IL10 for 48 h. To induce M2a and M2c macrophages, the M1 macrophages were also incubated with IL4 (100 ng/mL) and IL10 (100 ng/mL) cytokines for 48 h respectively. The transfected M1 macrophages were then fixed in 4% formalin, followed by blocking non-specific binding with bovine albumin serum (BSA) (3% w/v) at room temperature for 30 min. The cells were stained with Alexa Fluor 488-anti-mouse CD80, CD206 or CD163 antibodies for 1 h at room temperature at a dilution of 1:200. For analysis of CD44 expression, unpolarized J774A.1 cells were incubated with BSA (3% w/v) for 30 min, followed by staining with anti-mouse CD44 antibody for 1 h at room temperature. The cells were then washed thrice with PBS for FACS analysis in FL1 channel using a BD FACScalibur instrument (San Jose, CA). The data were analyzed using Cell-Quest Pro software.

### *In vitro* transfection studies

pDNA transfection studies were conducted to quantify the overexpression of IL4 and IL10 genes in macrophages after exposure to HA-PEI/pDNA NPs. Unpolarized macrophages (200,000 cells per well) were plated in 6-well plates were transfected with HA-PEI/pDNA-IL4 and HA-PEI/pDNA-IL10 NPs at a dosage of 20 *μ*g of pDNA per 200,000 cells. Naked pDNA and pDNA complexed with Lipofectamine® 2000 (a cationic lipid transfection vector) were used as controls. After 6 h incubation with HA-PEI/pDNA-IL4 and HA-PEI/pDNA-IL10 NPs, naked plasmid, pDNA-lipofectamine 2000, the samples were removed and replaced with fresh DMEM medium. At 12 h, 24 h, and 48 h post-transfection, the cells were harvested for RNA isolation, and expression levels of the IL4 and IL10 genes were quantified using quantitative PCR (qPCR) (LightCycler 480, Roche, Branford, CT). *β* -actin was used as a house keeping gene.

### *In vitro* polarization studies

The ability of HA-PEI/pDNA-IL4 and HA-PEI/pDNA-IL10 to modulate macrophage phenotype from M1 to M2 was assessed by measuring the change in the expression level of iNOS2 (M1 marker) and Arg1 (M2 marker) in J774A.1 macrophages after transfection with the NPs. J774A.1 macrophages (200,000 cells per well) were cultured in 6-well plates and stimulated to an M1 phenotype by overnight incubation with LPS (100 ng/mL) and IFN-*γ* (100 ng/mL). Subsequently, the M1 macrophages were transfected with HA-PEI/pDNA-IL4 and HA-PEI/pDNA-IL10 NPs at a dose equivalent to 20 μg of pDNA per 200,000 cells. After 6 h incubation, the cells were washed with PBS and cultured in complete DMEM medium. The cells were harvested at 0 h, 24 h, 48 h, and 72 h after the initial addition of the nanoparticles. The polarization effect was determined by quantifying the expression of iNOS2 (an M1 marker) and Arg1 (an M2 marker) using qPCR. *β* -actin was used as a house keeping gene.

### *In vivo* uptake of HA-PEI nanoparticles in peritoneal macrophages

All animal studies were performed according to an approved protocol by Institutional Animal Care and Use Committee (IACUC) at Northeastern University.

C57BL/6 mice (6–8 weeks old) were purchased from Harlan Laboratories (South Easton, MA). The mice were injected intraperitoneally with sterile Brewer-thioglycollate medium (2 mL, 4% w/v) to recruit macrophages to peritoneal cavity. At day 4 post-thioglycollate injection, Cy5.5 labeled HA-PEI NPs in PBS (0.5 mL of 1 mg/mL) was injected intraperitoneally. *In vivo* images at 6 h and 24 h post-injection of the NPs were acquired under an IVIS imaging system (Perkin Elmer, Hopkinton, MA). At 6 h and 24 h post-injection of the NPs, the mice were sacrificed for macrophage collection from mouse peritoneal cavity using cold PBS. The cell pellets obtained after centrifugation at 2000 rpm for 5 min were suspended in DMEM containing 10% FBS and 1% antibiotics and cultured in a Lab-Tek II chamber slide and a 6 well plate for 2 h at 37 °C and 5% CO_2_, followed by washing three times with PBS to remove non-adherent cells. Peritoneal macrophages were then blocked with BSA (3%) for 15 min at room temperature, followed by staining with FITC-conjugated anti-mouse F4/80 antibody (specific marker for mouse macrophages) and Alexa Fluor 488-anti-mouse CD44 at room temperature for 1 h. The cells were imaged under a Zeiss confocal microscope at an excitation wavelength of 488 nm for F4/80 antibody and 647 nm for Cy5.5 labeled HA-PEI NPs. FACS analysis for CD44 expression in peritoneal macrophages was performed in FL1 channel using a BD FACScalibur instrument.

### *In vivo* polarization of HA-PEI/pDNA NPs in C57BL/6 mice

C57BL/6 mice (6–8 weeks old, 20 g) purchased from Harlan Laboratories were injected intraperitoneally with sterile Brewer-thioglycollate medium (2 mL, 4% w/v). At day 3 post-thioglycollate injection, the mice were randomly divided into different groups (n = 4). Except for the control group, all mice received LPS (0.5 mg/kg) and IFN-γ (0.1 mg/kg) via IP administration. At 2 h post-LPS and IFN-γ injection, the mice were injected intraperitoneally with naked plasmid, and HA-PEI/pDNA NPs at 100 μg pDNA per mouse. At 48 h post-injection of pDNA, the mice were sacrificed for macrophage collection using cold PBS. Peritoneal macrophages were separated from the peritoneal exudate cells by magnetic beads conjugated anti-mouse Cd11b antibody using MACS Cell Separation kit (Miltenyi Biotec, San Diego, CA). Purified peritoneal macrophages were then used for RNA isolation using High Pure RNA isolation kit from Roche Applied Sc. (Indianapolis, IN) and cDNA synthesis using Verso cDNA Synthesis kit from Thermo Scientific Inc. (Waltham, MA). Expression levels of IL4, IL10, TNF-α, iNOS, and Arg genes were quantified using qPCR. *β* -actin was used as a house keeping gene. For FACS analysis of M2 surface markers in the peritoneal macrophages, purified peritoneal macrophages were stained with anti-mouse PE-F4/80, Alexafluor 488-CD206, and Alexafluor 488-CD163 antibodies. The cells were then washed thrice with PBS for FACS analysis in FL1 and FL2 channel using a BD FACScalibur instrument.

### *In vivo* anti-inflammatory effect of HA-PEI/pDNA NPs in C57BL/6 mice

C57BL/6 mice (6–8 weeks old, 20 g) purchased from Harlan Laboratories were injected intraperitoneally with sterile Brewer-thioglycollate medium (2 mL, 4% w/v). At day 3 post-thioglycollate injection, the mice were injected intraperitoneally with naked pDNA or HA-PEI/pDNA NPs at 100 μg pDNA per mouse. After 24 h of pDNA injection, the mice were received LPS (1 mg/kg) via IP administration. At 24 h post-LPS injection, the mice were sacrificed for collection of blood, peritoneal fluid and peritoneal macrophages. Peritoneal fluid was collected from peritoneal cavity after IP injecting 1 mL of PBS to the mouse peritoneal cavity. Peritoneal macrophages were separated from the peritoneal exudate cells by magnetic beads conjugated anti-mouse Cd11b antibody using MACS Cell Separation kit (Miltenyi Biotec, San Diego, CA). Purified peritoneal macrophages were then used for measurement of mRNA expression of IL4, IL10, TNF-α, IL-1β genes by qPCR. *β* -actin was used as a house keeping gene. Protein levels of IL4, IL10, TNF-α, IL-1β in serum and peritoneal fluid were quantify by enzyme-linked immunosorbent assay (ELISA) using mouse-specific ELISA kits for IL4, IL10, TNF-α, and IL-1β purchased from Ebioscience (San Diego, CA).

### Data analysis

Data were expressed as mean ± standard deviation. Statistical significance was determined by one way analysis of variance (ANOVA) tests using SPSS (version 22.0). LSD post hoc multiple comparison tests were used to compare statistical significance at a 5% probability level. A probability (p) of less than 0.05 was considered statistically significant.

## Additional Information

**How to cite this article**: Tran, T.-H. *et al*. Modulation of Macrophage Functional Polarity towards Anti-Inflammatory Phenotype with Plasmid DNA Delivery in CD44 Targeting Hyaluronic Acid Nanoparticles. *Sci. Rep*. **5**, 16632; doi: 10.1038/srep16632 (2015).

## Supplementary Material

Supplementary Information
