# Supplementary material for: Modulation of Macrophage Functional Polarity towards Anti-Inflammatory Phenotype with Plasmid DNA Delivery in CD44 Targeting Hyaluronic Acid Nanoparticles
Source: Sci Rep. 2015 Nov 18;5:16632. doi: 10.1038/srep16632 (PMC4649614; doi:10.1038/srep16632)
Supplement: Supplementary Information [file srep16632-s1.doc]

**Supporting Information**

**Modulation of Macrophage Functional Polarity towards Anti-Inflammatory Phenotype with Plasmid DNA Delivery in CD44 Targeting Hyaluronic Acid Nanoparticles**

**Thanh-Huyen Tran, Ruchir Rastogi, Juili Shelke, Mansoor Amiji***


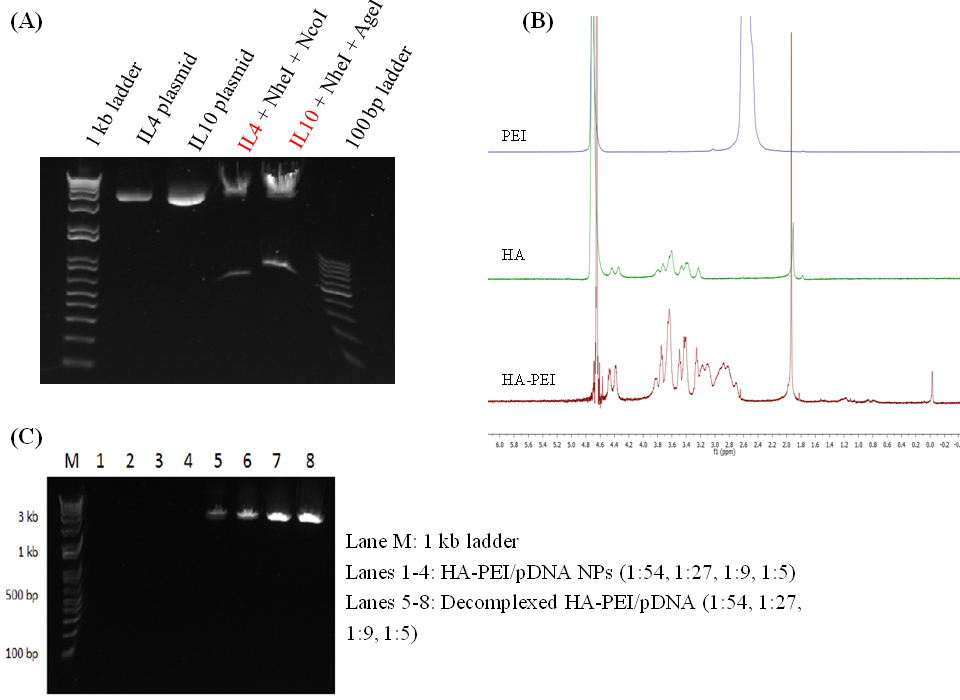


**Figure S1.** (A) Undigested and double digested pDNA-IL4 and pDNA-IL10 with restriction enzymes NheI and NcoI, or NheI and AgeI, respectively. Fig. S1A showed bands of 500 and 600 bp in lanes 4 and 5 indicating the presence of IL4 and IL10 genes within the plasmid. (B) ^1^H NMR spectra of HA, PEI and HA-PEI conjugate dissolved in D_2_O. (C) Electrophoretic retardation analysis of pDNA-encapsulated HA-PEI nanoparticles at different mass ratios (54:1, 27:1, 9:1, and 5:1) and release of intact pDNA by polyacrylic acid decomplexation.

**Figure S2**: Expression of IL4 and IL10 genes in J774A.1 upon transfection with blank HA-PEI for 12h, 24h, and 48h at the same concentration used for delivery of pDNA-IL4 and IL10 (180 μg/mL). No significant change in the IL4 and IL10 gene expression in J774A.1 cells upon treatment with blank HA-PEI at the tested concentration.


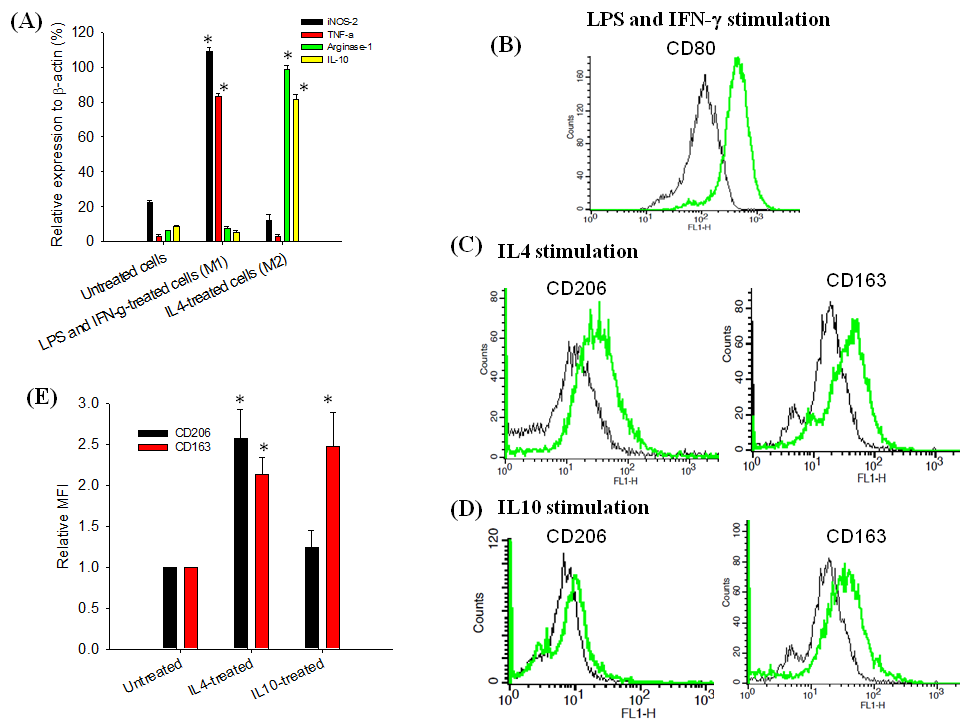


**Figure S3.** (A) Relative expression of M1 genes (TNF-α and iNOS) and M2 genes (Arg and IL10) in J774A.1 macrophages stimulated with LPS combined with IFN-γ, and IL4 cytokine for 16 h. (B) CD80 expression in J774A.1 macrophages stimulated with LPS and IFN-γ for 16 h. CD206 and CD163 expression in J774A.1 cells stimulated with IL4 cytokine (C) and IL10 cytokine (D) for 16 h. (E) Relative mean fluorescence intensity (MFI) compared to untreated cells. Black line-untreated macrophages; green line-macrophages stimulated with different cytokines. n=3, p<0.05 compared to untreated macrophages.


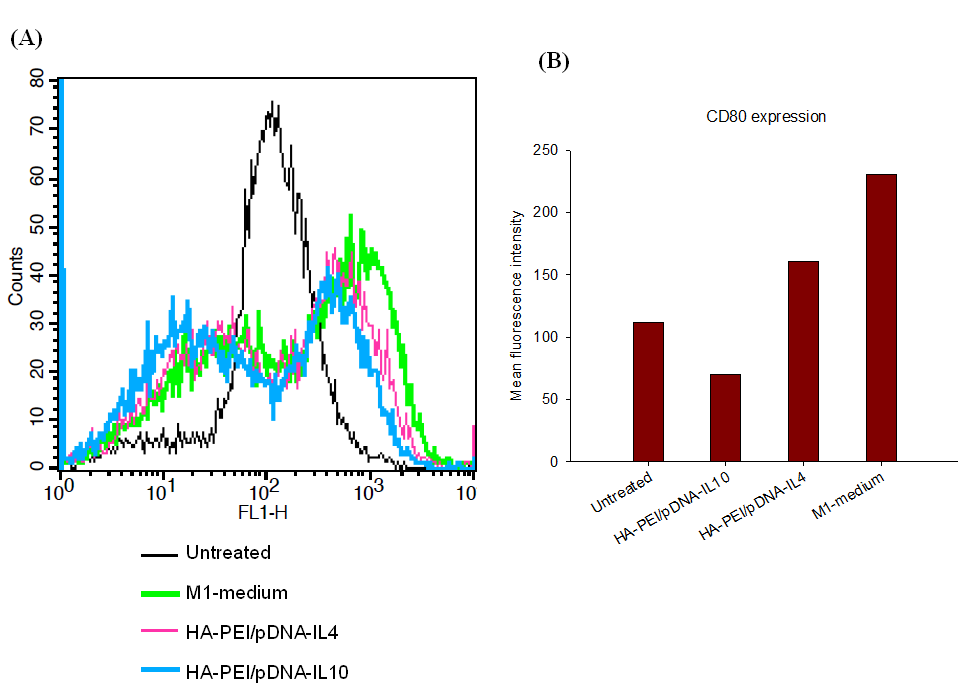


**Figure S4.** (A) CD80 expression and (B) Mean fluorescence intensity of LPS and IFN-γ stimulated J774A.1 macrophages (M1) incubated with complete medium or transfected with HA-PEI/pDNA-IL4 and HA-PEI/pDNA-IL10.


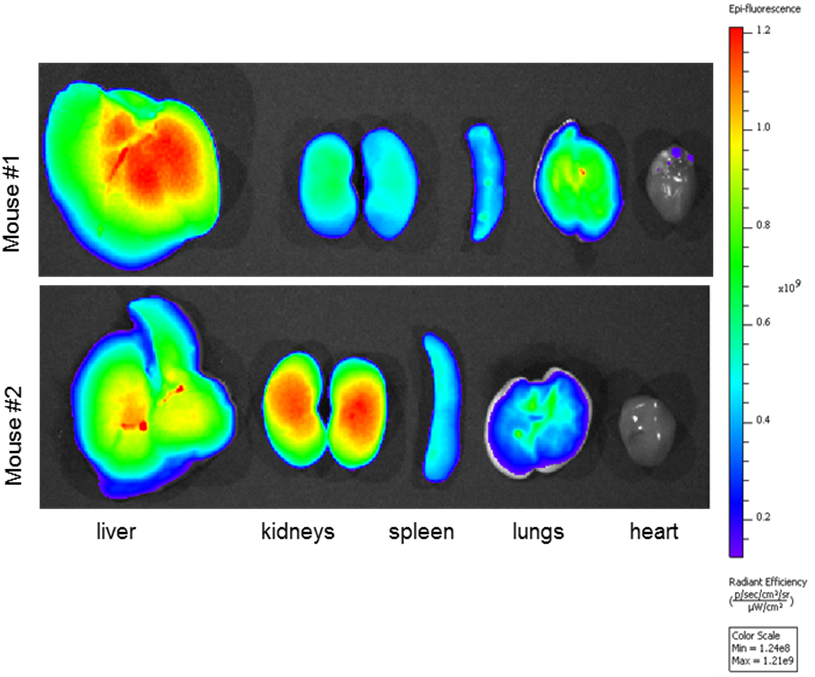


**Figure S5.** Tissue near-IR fluorescence imaging of Cy5.5-conjugated HA-PEI nanoparticles upon IP administration in C57BL/6 mice.

**Results**

The ^1^H NMR spectrum of HA-PEI as compared to that of HA and PEI has both a methyl peak of acetoamide group of HA at 1.9 ppm and a PEI peak at 2.5-3.2 ppm shifted from 2.5 ppm (**Fig. S1B**) as reported in previous studies . The result indicated successful conjugation of PEI to HA with 32% PEI modification as calculated by ^1^H NMR spectroscopy.

To investigate loading capacity of HA-PEI for pDNA-IL4 and pDNA-IL10, the plasmid in solution was physically mixed with HA-PEI solution in PBS at different polymer-to-pDNA weight ratios (54:1, 27:1, 9:1, and 5:1), and run on a 0.8% agarose gel. **Fig. S1C** showed the absence of plasmid band at all ratios when pDNA was encapsulated in the HA-PEI NPs (lane 1-4), indicating that pDNA was completely encapsulated into HA-PEI even at 5:1 ratio.

1. Jiang G*, et al.* Hyaluronic acid-polyethyleneimine conjugate for target specific intracellular delivery of siRNA. *Biopolymers* **89**, 635-642 (2008).

2. Mahor S, Dash BC, O'Connor S, Pandit A. Mannosylated polyethyleneimine-hyaluronan nanohybrids for targeted gene delivery to macrophage-like cell lines. *Bioconjugate chemistry* **23**, 1138-1148 (2012).
